# The niche reduction approach: an opportunity for optimal control of infectious diseases in low-income countries?

**DOI:** 10.1186/1471-2458-14-753

**Published:** 2014-07-25

**Authors:** Benjamin Roche, Hélène Broutin, Marc Choisy, Sylvain Godreuil, Guillaume Constantin de Magny, Yann Chevaleyre, Jean-Daniel Zucker, Romulus Breban, Bernard Cazelles, Frédéric Simard

**Affiliations:** UMMISCO (UMI 209 IRD-UPMC), Centre IRD-France Nord, 32, avenue Henry Varagnat, 93143 Bondy, Cedex, France; UMR MIVEGEC (IRD 224-CNRS 5290-UM1-UM2), Montpellier, France; Oxford University Clinical Research Unit, Hanoi, Vietnam; INSERM U1058 & Department of Bacteriology-Virology CHU Arnaud de Villeneuve, Montpellier, France; UMR CNRS 7030 & Université Paris 13, Villetaneuse, France; Institut Pasteur, UEME, Paris, France; UMR 7625 UPMC-CNRS-ENS, Paris, France

## Abstract

**Background:**

During the last century, WHO led public health interventions that resulted in spectacular achievements such as the worldwide eradication of smallpox and the elimination of malaria from the Western world. However, besides major successes achieved worldwide in infectious diseases control, most elimination/control programs remain frustrating in many tropical countries where specific biological and socio-economical features prevented implementation of disease control over broad spatial and temporal scales. Emblematic examples include malaria, yellow fever, measles and HIV. There is consequently an urgent need to develop affordable and sustainable disease control strategies that can target the core of infectious diseases transmission in highly endemic areas.

**Discussion:**

Meanwhile, although most pathogens appear so difficult to eradicate, it is surprising to realize that human activities are major drivers of the current high rate of extinction among upper organisms through alteration of their ecology and evolution, i.e., their “niche”. During the last decades, the accumulation of ecological and evolutionary studies focused on infectious diseases has shown that the niche of a pathogen holds more dimensions than just the immune system targeted by vaccination and treatment. Indeed, it is situated at various intra- and inter- host levels involved on very different spatial and temporal scales. After developing a precise definition of the niche of a pathogen, we detail how major advances in the field of ecology and evolutionary biology of infectious diseases can enlighten the planning and implementation of infectious diseases control in tropical countries with challenging economic constraints.

**Summary:**

We develop how the approach could translate into applied cases, explore its expected benefits and constraints, and we conclude on the necessity of such approach for pathogen control in low-income countries.

## Background

### Successes and failures of pathogen control

On June 28^th^ 2011, the Food and Agriculture Organization for the United Nations (FAO) has officially announced the eradication of rinderpest from the surface of the globe, 10 years after the last recorded case [1]. Thirty years had passed since the previous, and then only, global eradication of an infectious pathogen, namely the smallpox [2]. These examples are the most striking in an array of spectacular achievements of public health interventions coordinated by WHO for the last 60 years. Indeed, numerous pathogens have been eliminated in large areas, such as malaria [3] and plague [4] in Western countries, or yellow fever in Latin America [5].

Meanwhile, in many tropical countries, infectious diseases continue to exert a major toll on human populations and directly contribute to poverty and economic instability [6]. Pathogen richness is indeed generally higher in the tropics than in temperate areas [7] and in many cases, vector biodiversity further adds to the complexity of the transmission system [8]. Emblematic examples include malaria in Africa [9] where both the parasite and its mosquito vector species have now developed high levels of resistance to the most widely used drugs and insecticides, further jeopardizing control as well as elimination efforts [10, 11]. Yellow fever is another frustrating example where the pathogen remains endemic in many countries [12] despite the availability of an easily deliverable (lyophilized) and affordable vaccine [13] and because of uncontrolled sylvatic transmission cycles [14] allowing virus maintenance In turn, the current eradication campaign against poliomyelitis illustrates a tradeoff between limited available economic resources and their allocation to solve the myriad of problems that conflict or post-conflict countries have to confront without functioning governance and public administrations [15]. Moreover, in Pakistan and Afghanistan, although funding for polio eradication might not be an issue owing to substantial input from international and non governmental agencies, societal concerns about vaccination as well as the circulation of vaccine-derived polio viruses may represent the biggest roadblocks to successful eradication [16, 17]. Last but not least, the HIV pandemic, as well, still defies efficient control because 80% of the infected individuals live in countries where current antiretroviral therapies remain prohibitively expensive [18].

These issues do not concern only elimination and eradication programs, but also public health strategies aiming at reducing pathogen burden in general. Indeed, our current world is increasingly interconnected [19, 20] and thus, failures to manage local epidemics may rapidly convert into global threats, as demonstrated by the SARS, Chikungunya, H5N1 and H1N1 pandemics [21–23] during the last decade and currently by H7N9 [24]. There is thus an urgent need to explore new approaches for the development of affordable and sustainable disease control tools and strategies applicable on a global scale and particularly in low-income countries.

## Discussion

Whereas most pathogens appear so difficult to control, to eliminate and/or to eradicate, evidence is accumulating that human-induced environmental changes and habitat destruction impact the ecology and evolution of upper organisms and thus contribute significantly to the current high rate of extinction [25]. Today, it is largely accepted that an alteration of the niche of a species (*i.e.,* its position relative to available resources and competitors present in the environment) is an efficient driver to extinction if the species cannot evolve fast enough to fill another niche or to adapt to the altered one (*e.g.,* 82% of endangered bird species are weakened by niche alteration, especially habitat loss [26]). From this perspective, recent advances in ecology and evolutionary biology of pathogens [27–31] provide unprecedented opportunities to build on a new paradigm in the way pathogen control is being devised and implemented. By promoting research for manipulation of pathogens niche as an efficient and effective means for their control, we advocate the application, in public health, of the opposed methodologies usually envisioned to rescue endangered species in order to reach the opposite target of conservation biology, *i.e.,* the extinction (or eradication as it is often referred to in epidemiology). Then, we call for integrating *disease control* within a broader perspective, focusing on the *reverse conservation biology* of pathogens.

### What is a pathogen niche?

Ecological niche is a key concept in ecological literature, for which many different definitions can be considered [32, 33]. From a very general point, we can define the niche of an organism as the position of this organism within its environment, available resources and competitors. Then, it is characterized by all the conditions required to sustain a viable population of the organism, in space and time. For instance, the “fundamental” niche of African lions contains grasslands and savannah prairies (their habitats) as well as wildebeest and zebras (their resources). However, African lions “share” their habitats and their resources with other predators such as hyenas or wild dogs, yielding a “realized” niche that is the “fundamental” one constrained by the presence of competitors.

**Figure 1:**
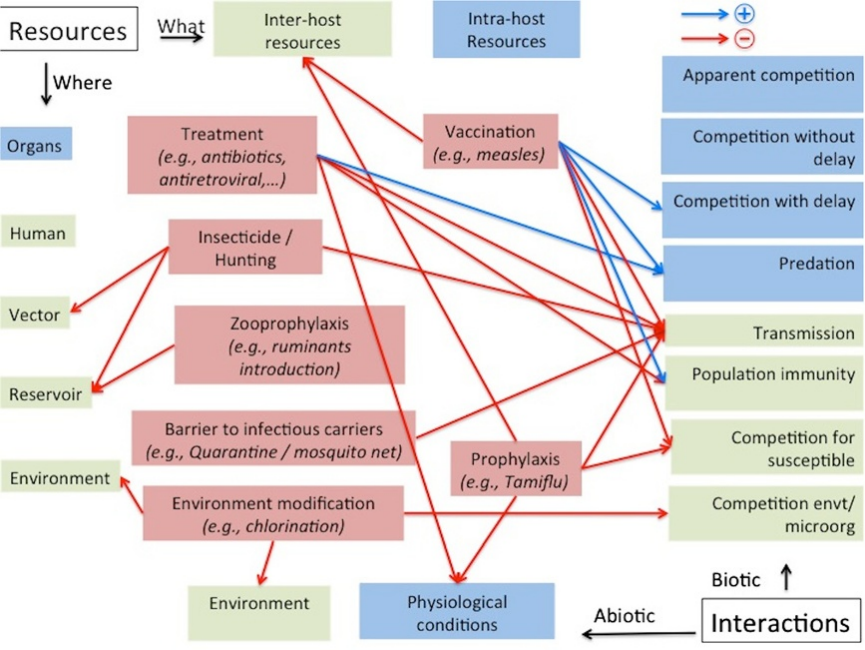
**Different components of the niche of a human pathogen and how it can be used for its control.** The green and blue boxes represent resources and interactions situated at an inter- and intra-host level, respectively. The red boxes depict the main current methods used in public health and their positive or negative impact on niche components (*e.g.,* vaccination decreases number of susceptible individuals, and increases population immunity).

Then, pathogens, just like any organism, may have a realized and a fundamental niche [34]. Since we aim at applying pathogens niche manipulation to propose new strategies in public health, all the potential resources and interactions have to be considered. During the last decades, the accumulation of ecological and evolutionary studies focusing on infectious diseases has shown that the niche of a pathogen holds more dimensions than just the host immune system targeted by vaccination. More specifically, the niche of a pathogen encompasses diverse intra- and inter-host levels (Figure 1). Consequently, its boundaries and evolution will depend upon a number of biotic and abiotic interactions that go far beyond competition for the resource in susceptible hosts, the presence of pathogens in the environment or the activation of the immune system machinery in the host and/or the vector.

In many cases, human pathogens are also able to infect and develop in other vertebrate hosts and most human infectious diseases have a zoonotic transmission cycle [35], in which the animal reservoir is fundamental to consider. In addition, vector-borne pathogens do possess a required step of development within their arthropod vector species [36] while environmentally-transmitted pathogens may persist during a significant period of time outside the host [37]. Then, a pathogen may have biotic interactions with many upper organisms and abiotic interactions with environment outside the host, including in the case of vector-borne diseases where the vector is an ectotherm arthropod.

Within-host pathogen space is not that large either. Evidences are accumulating that pathogens can compete within the same host. This competition can take several forms, through competition for resources such as red blood cells between different species of *Plasmodium* [38] or specialized cells within an organ like in the interaction between influenza viruses and pneumococcal bacteria in lungs [39]. Pathogens interactions are also mediated by the immune system, such as in the case of malaria and helminths [40] where each pathogen activates different and inter-dependent immune paths (Th1 and Th2). It is also the case for HIV that depresses the whole host immune system therefore fostering the development of opportunistic infections, especially to *Mycobacterium tuberculosis* (TB) where individuals infected by HIV are 20 to 30 times more likely to develop an infection [41], a phenomenon enhanced by a higher fatality rate during treatment of co-infected patients. Finally, the within-host conditions also play an important role [42], through for instance malnutrition [43] or vaccine history that alters immune system efficiency [44].

The different layers that make up a pathogen’s niche, involving various intra- and inter-host levels, impact the infection process at two scales: host infection (colonization) and within-host invasion (within-host pathogen replication). Then, it follows that the initial and fundamental resource for a pathogen is not only the availability of susceptible hosts to infect but also the ability of hosts to sustain a sizable pathogen load.

### Public health strategies and pathogen’s niche: Applying “reverse conservation biology” to pathogens

#### Classic control measures and pathogen’s niche

**Figure 2:**
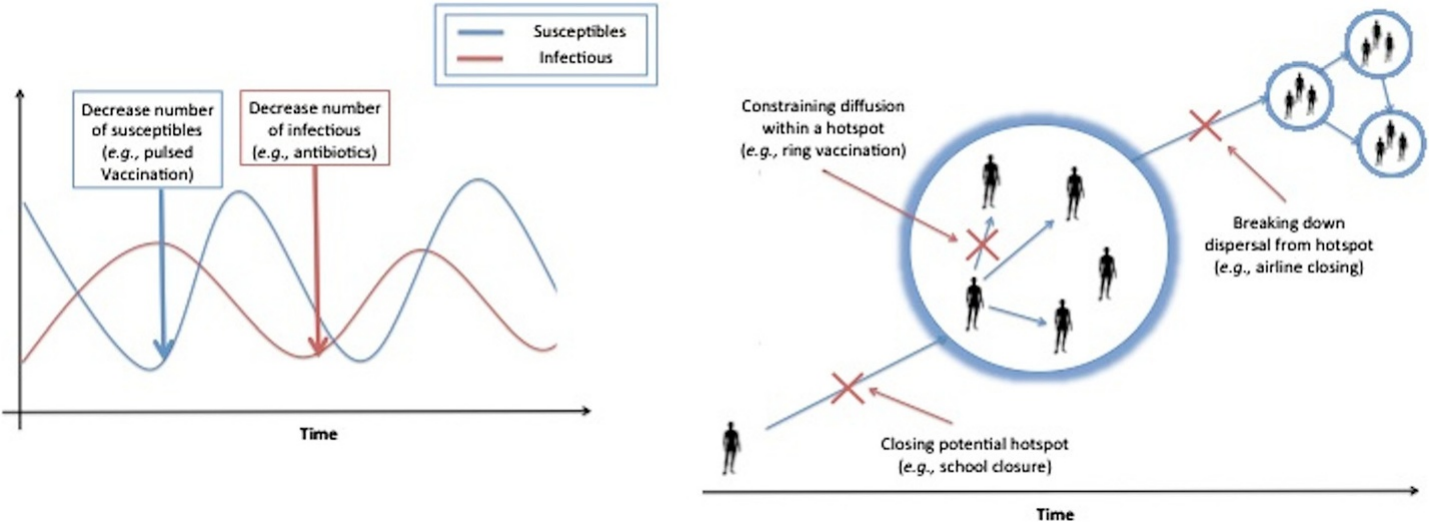
**Examples of the evolution of a pathogen’s niche in space and time and how it can be manipulated for control.** (Left ) Illustration of fluctuations in the number of susceptible and infectious individuals with time and an example of optimized pathogen control combining pulsed vaccination of susceptible hosts and timely treatment of infectious ones. (Right) The host geographic structure can be used and manipulated to stop a pathogen spill-over by restricting opportunities for migration between transmission hotspots where resources (i.e., susceptible hosts) are plentiful.

Following the above reasoning, most public health strategies for pathogens control focuses on decreasing the susceptible host population, especially through prophylaxis (drugs or preventive vaccination). Figure 1 shows that this reduction results mainly from the targeting of biotic interactions. Nevertheless, ecological theory tells us that pathogen niche may also fluctuate in space and time. Thus, this decrease in the susceptible population can be organized more efficiently, by considering pathogen’s dynamics, i.e. its natural fluctuation in space and time (Figure 2), opening opportunities to widen the impact of preventive strategies while facilitating their implementation through targeted actions in space and time. This is already applied in some specific situations, especially sporadic outbreaks of a threatening pathogen such as meningitis [45], but this opportunity should be considered for endemic pathogens as well.

#### How can the niche concept improve public health strategies design and implementation?

Ecological and evolutionary literature on which the niche concept relies is rich in lessons for devising and combining public health strategies towards maximizing their expected impact on pathogen transmission. A key observation is that organism extinction usually stems from the simultaneous alteration of several components of its niche [46], such as resource availability [47] or competition pressure [48]. This empirical and theoretical framework, supported by many different kinds of organisms [49–52] provides crucial insights to envision a new paradigm in public health and infectious diseases management. Indeed, these results suggest that combining public health measures that target different components of a pathogen’s niche is expected to constrain more efficiently pathogen’s survival and transmission.

As a prerequisite to combining different strategies for pathogens control in the field, one will need to assess whether the effects of the different strategies applied at the same time combine additively or not, in order to identify the most relevant and synergistic combinations. Moreover, when the net effect of strategies combination is more than the simple addition of each strategy considered separately, the approach could be a very strong asset for public health program management. Indeed, with a given financial resource, it may be more efficient to dedicate a proportion of this money to one control measure, e.g., targeted vaccination, and the rest into another control measure, such as vector control, rather than 100% in either of the two. This clearly calls for theoretical studies that will inform on the expected outcomes of the different control methods, used alone and in combination, as well as foster the development of a modeling framework to identify the most suitable strategies admixtures that will allow optimal efficiency in terms of prevention and mitigation of infections. Such modeling framework could benefit from the last methodological developments in mathematical epidemiology [30] and optimization theory [53].

### Recent ecological knowledge relevant to public health management?

Recent ecological and evolutionary studies of infectious diseases have shed light on examples of pathogen niche alteration with an impact on pathogen fitness, transmission and/or virulence. For example, the presence of competing pathogens is especially threatening for organism survival, such as in the case of co-infection with *Plasmodium falciparum* and helminths that can decrease malaria severity in humans [54]. Similarly, maternally inherited endosymbiotic bacterial *Wolbachia* infections were shown to directly reduce the susceptibility of insects to infection with a range of insect and human pathogens [55], above and beyond their impact on vector’s life history traits relevant to vector capacity and transmission (*e.g.,* blood feeding frequency, longevity, fecundity…). Niche alteration can also result from a temporary mismatch between pathogens and hosts abundance. Many pathogens reproduce at a given period of time when resources are abundant and of good quality. It is known, for instance, that apparent competition can take place between two pathogens because one removes susceptible hosts to the other one, such as in the case of measles and whooping-cough [56]. This case definitely represents two pathogens with a niche overlap.

### Current control programs and the pathogen niche approach: turning theory into practice

#### Current public health programs: where do we stand?

Current public health programs are highly heterogeneous in their accounting for the multi-dimensionality of the ecological niche of their target pathogens, ranging from single specific approaches to integrated control initiatives. Dengue and trachoma control are emblematic examples of the extremes in this continuum. In the absence of a vaccine and efficient drugs, dengue control chiefly relies on vector control, aiming at reducing mosquito abundance to reduce disease transmission. On the other hand, the SAFE WHO program [57], targeting the bacterium *Chlamydia trachomatis* that causes trachoma uses a combination of surgery, antibiotics, facial cleanliness and environmental improvement to track the pathogen in different environments. However, it is estimated that dengue viruses still infect around 100 million peoples each year [58]. Similarly, 18 million of active trachoma cases have been observed in 2012 [59], highlighting that current public health strategies can still be improved.

#### How can the pathogen niche approach improve current public health programs?

The niche approach could improve public health programs implementation and strategy development in several ways. First, as highlighted above for measles [60] or polio [15], the majority of the current strategies that mainly focus on one dimension of the pathogen niche require highly effective and fast-acting control tools, and a high coverage of target groups/populations. This could be unrealistic is some settings (see above). In this context, the ecological theory suggests the importance of multiplying targets, rather than increasing selective pressure on a single mechanism, in order to circumvent the apparition of resistances. As an example, Dengue control mainly focuses on vector control while other strategies, such as the use of *Wolbachia* that constrain virus development in its mosquito vector or the currently-tested vaccine are emerging and should prompt a new approach to dengue control programs in a near future. For diseases where vaccine is the only control possibility, an immunization strategy finely-tuned to the pathogen spatio-temporal dynamics could still improve significantly its effectiveness. First attempts considering only temporal dimension have already shown the potential of such approach [61] and should be extended to the spatial dimension, by focusing on disease (or transmission) hotspots.

Second, for the current control strategies that already focus on several targets within the pathogen’s niche, for which the trachoma control program SAFE represents an excellent example, a major insight from the pathogen’s niche approach is the importance of considering the temporal and spatial dimensions. Indeed, the necessity to include spatio-temporal dynamics of pathogens to understand local transmission, as well as efficiency of control programs, has been extensively demonstrated [62, 63]. As previously exposed, the pathogen niche moves in space and time. This implies that a period of time exists where control could be most efficient, which generally precedes the observation of optimal conditions for pathogen transmission. As an example, if vector abundance presents a seasonal pattern, it could be more efficient to enforce vector control strategies when abundance is low as vector populations might then be more amenable to control in otherwise adverse environmental conditions.

Finally, ecological theory tells us that the evolutionary dimension is also crucial to consider. The sequential usage of different antibiotics (for trachoma) or insecticides/larvicides (for dengue vectors) with different modes of action for example, is an efficient way to reduce the risk of emergence of resistance, and to mitigate its spread, therefore promoting sustainability. The concept of Late-Life-Acting insecticides *i.e.,* compounds or organisms that slowly kill the arthropod vector such that it dies after reproduction but before it is able to transmit the pathogen also directly stems from ecological and evolutionary biology theory, and appears as a promising tool for sustainable transmission control [64].

Moving from the concept of pathogen niche reduction to implementation of a sustainable control strategy in the field requires consolidated trans-disciplinary collaboration among scientists and will rely on endorsement by all stakeholders at the political and societal levels (e.g., disease control programs, ministries, traditional and state representatives,…). While modeling will provide important insights to carefully tailor these new public health strategies, current methods used in public health, such as controlled field trials and careful monitoring of epidemiological and ecological outcomes, remain essential to validate models and set the ground for scaling up. Moreover, in many cases implementation of the niche reduction approach should build on existing control programs and on strategies that are already implemented in the field, and foster the development of truly integrative control strategies that will take into account the evolutionary dynamics of the pathogen.

#### Creating synergy from improvements produced by the pathogen niche approach

While the benefits of using evolutionary theory to improve current diseases control tools can be seen as intuitive and are well-known to the public health community workers, the major breakthrough due to the pathogen niche approach is synergy stemming from the combination of such improvements, and the ability to capitalize on them. For this to occur, careful tailoring of implementations in space and time will be required. For example, identifying the key period(s) and areas where different control methods should be applied and combined with the highest expected efficacy could be crucial for trachoma control, ensuring rational control at the lowest cost.

A natural candidate disease for this approach could be dengue. Indeed, a vaccine could be released reasonably soon which targets a completely different compartment of the virus niche than current vector control programs. We are then in a context where combining vector control and prophylactic drugs when vector abundance is low (i.e., either naturally, through seasonality of vector abundance or artificially, through the use of vector control strategies) can yield an extremely efficient pathogen control. The short infectious period of dengue suggests that this combination has to be applied during a short period of time, which is another benefit from this approach.

#### Control programs using pathogen niche in action: success stories

An emblematic example of such concept in action is malaria elimination in Italy during the first half of the twentieth century [65]. Patients were mass-treated with chloroquine during wintertime when mosquitoes can only survive indoors and were therefore particularly amenable to elimination through spraying of residual insecticides. In this case, chloroquine has decreased abundance of susceptible humans and insecticides have annihilated vector populations at a time when they were already at their nadir.

Another example of working strategies that target different components of the pathogen’s niche is the case of tri-therapies to treat HIV [18]. Indeed, patient well-being has improved significantly in recent years thanks to the association in a single treatment dose of different molecules targeting different parts of the viral cycle, including protease inhibitors that block virus replication and reverse-transcriptase inhibitors that constrain infections within cells.

### Limitations and potential drawbacks of the pathogen niche approach

The challenge of the niche approach implementation is double. First, it relies on the availability of several control tools with different targets that can be implemented together at the same time in the same place, or sequentially in time and space. Pathogens and vectors are known to be extremely plastic in their behavior with a high adaptive potential. Failures in implementation or incorrect planning might result in pathogen escape and niche shift with unforeseen consequences for human health and the environment. The second challenge therefore resides in the ability of public health managers to act fast and implement several control strategies together locally, within a short time-frame, but also to repeat such actions during several years in order to ensure an efficient pathogen control over the long-term. From the point of view of economic resources, the costs can be decreased quite significantly (lower level of drug use, less storage, shortening the necessity of cold chain,…), but the logistics and organization could be more complex to sort out.

These drawbacks are important and cannot be neglected. They especially highlight that the niche approach should be applied only to pathogens for which the ecological and evolutionary dimensions are sufficiently documented and achieved large scientific consensus.

### Adopting a niche approach: a relevant thought for pathogen control in low-income countries

We believe that the niche approach is particularly promising for countries with challenging economic constraints where traditional approaches for diseases control could not be implemented successfully and/or maintained over time mainly due to financial shortcomings. Indeed, as exemplified above, niche alteration has already been successfully applied. Nevertheless, these first successes are encouraging and clearly deserve to be continued in this specific context of economic constraints. Moreover, most of the pathogens affecting human populations are now studied through the lens of ecology and evolutionary biology, extending dramatically the possibilities on which public health strategies can rely. This new and abundant literature documenting each part of the pathogen’s niche should now be integrated into policy decisions on public health strategies.

The main benefit of such an approach is the opportunity to shift from a public health strategy working on a long time period to a public health strategy that targets specific components of the disease system during a specific period within a specific area. If we place this perspective in the case of low-income and emerging countries where disease elimination is not a priority, this approach can increase political interest because it provides opportunities to (i) decrease the cost of such public health programs and (ii) the period of time where some infrastructures have to be activated.

Thus, carefully tailoring public health interventions to the pathogen niche may provide opportunities for improving their success by enhancing control effectiveness while maintaining low cost. We believe that mathematical modeling, extensively used into the study of ecology and evolution of infectious diseases to connect theoretical mechanisms and observed patterns, is a privileged tool to design innovative strategies finely tuned to different pathogens. If such low-cost solutions, direly needed in economically constrained countries, can be identified and proved successful from both a theoretical and applied standpoint, it could be extended to other parts of the World and applied at a global scale.

## Summary

### In public health, it is sometimes better to do nothing than just a little

In resource-poor settings, public health officers may only have access to a limited arsenal and supplies for diseases control such as curative treatment, or prophylactic measures (e.g., vaccines, antibiotics or Insecticide Treated Nets). Pursuing the goal of protecting local populations according to the protocols used in the developed world, officers may use available resources with poor efficacy and no clear assessment of the consequences. For example, inappropriately managed, vaccination against childhood diseases may be detrimental in the long term because it may postpone the mean age of infection to age classes where the disease is more severe. An example stemming from the history of malaria control is the poor management of therapy (*e.g.,* chloroquine) and/or insecticides (*e.g.,* DDT and pyrethroids) that yielded widespread resistance in most, if not all, major African countries.

### But doing nothing is never acceptable

**Figure 3:**
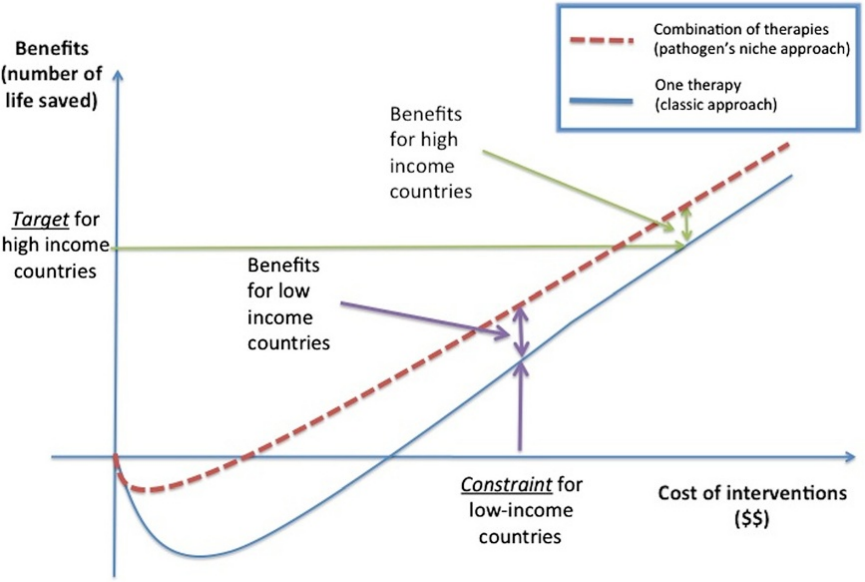
**Envisioned benefits of the pathogen niche approach.** Public health strategies are generally conditioned by an economic constraint in low-income countries whereas they target maximal burden alleviation for high-income countries. Methods for pathogen control are known to make things worse than doing nothing if coverage is below a given threshold (*e.g.,* insufficient vaccine uptake against childhood infections may postpone infections to teenage years when the disease has higher morbidity). Combining different methods for pathogen control (*e.g.,* vaccination tuned along the spatio-temporal dynamics of the pathogen in addition to quarantine) can decrease the risk of unexpected backfires of public health programs and offer greater public health benefits for similar cost.

Infectious diseases control must be applied globally, including in resource-poor countries. Ecological and evolutionary theories can inform Public Health and policy makers on which component(s) of the pathogen’s niche are the most appropriate and promising targets (Figure 3). Grand strategies of how to impact on the pathogen niche in various ways simultaneously should be investigated in the light of financial constraints and potential negative consequences for pathogen niche shifts and public health. Combining recent advances in mathematical modeling of infectious diseases, operational research and financial optimization methodologies, scientists would arm public health practitioners to prepare effective and realistic solutions.

It is worth pointing out that ecological niche theory is not the only concept from ecology and evolutionary biology that may help envision innovative public health strategies. For instance, invasion biology [66], exploring specific traits that lead species to become invasive or pests, might provide relevant insight and foster the development of a suitable conceptual framework for devising strategies aiming at preventing novel pathogens emergence and spread, especially during this current era of emerging and re-emerging infections [67]. Despite developing such ideas is outside the scope of this paper, we believe that such translation of ecological and evolutionary concepts into public health should become a research priority.

The approach should cause a paradigm shift in public health, since we argue that a strong, but short, control program does not fit with low-income countries settings. Instead, altering the pathogen’s niche, *i.e.,* changing significantly its environment in a permanent way, can lead to a better control in the long term. Moreover, it has been suggested that pathogen elimination in large territories are generally stable over time, i.e., a pathogen that has been eliminated does not easily re-emerge [68]. Then, while classical tools such as drugs, therapies or vaccines are definitely required to fight these pathogens, using them within a pathogen’s niche approach should allow a sustainable pathogen control in those specific areas, increasing the probability of elimination.
